# The Relationship Between Health Management and Information Behavior Over Time: A Study of the Illness Journeys of People Living With Fibromyalgia

**DOI:** 10.2196/jmir.5309

**Published:** 2016-10-25

**Authors:** Annie T Chen

**Affiliations:** ^1^ University of Washington School of Medicine Department of Biomedical Informatics and Medical Education Seattle, WA United States

**Keywords:** health knowledge, attitudes, practice, information seeking behavior, information use, consumer health information, chronic disease, fibromyalgia

## Abstract

**Background:**

Over the course of a chronic illness, patients face many challenges, including understanding what is happening to them and developing an effective strategy for managing illness. While there is existing literature concerning how people seek health-related information and cope with chronic illnesses, there is a need for additional research on how information affects patients’ understandings of their illness, and how changes in this understanding affect their health management strategies over time.

**Objective:**

This study examined how health management, information seeking, and information consumption and use processes are related throughout an illness.

**Methods:**

A diversified recruitment strategy involving multiple media channels was used to recruit participants for an interview study. During the interviews, participants were asked to draw an “illness journey” timeline. The data were analyzed using a qualitative approach drawn from Interpretative Phenomenological Analysis and Grounded Theory.

**Results:**

The study identified four main health management features of illness journeys: onset, progression toward diagnosis, acceptance, and development of an effective management strategy. The study then focused on how information seeking changes over illness journeys, particularly in terms of a transition from active information seeking to monitoring with intermittent focused searching. Last, the paper describes the information consumption and use processes that patients engaged in throughout their journey.

**Conclusions:**

This study makes three important contributions to the field. First, it presents an integrated conceptualization of how health management and information behaviors are related on illness journeys. Second, it adds to our existing knowledge on health literacy and self-management of chronic illness. Third, the study has implications for health interface design.

## Introduction

Over the course of a chronic illness, patients face challenges on many fronts. On a basic level, they endeavor to understand what is happening to them and deal with their illness. This may include navigating the health care system and understanding their medication regimen. They interact with information that may change their abilities to engage in these behaviors and make health decisions. While existing literature has investigated how people seek health-related information, there is a need for additional research on how information facilitates changes in patients’ understanding of their health, which may in turn lead to long-term changes in health management.

This study investigated the relationship between information and health management of those with a chronic condition—fibromyalgia. Fibromyalgia is a complex, poorly understood condition characterized by chronic widespread pain, joint stiffness, and systemic symptoms (eg, mood disorders, fatigue, cognitive dysfunction, and insomnia) [1-3]. Due to the diversity of symptoms and problems that patients experience, fibromyalgia has an impact on multiple facets of patients’ lives [4-6].

Because fibromyalgia patients often appear healthy and their symptoms are invisible, patients continually struggle with stigma, social isolation, and a search for legitimacy [7-10]. In addition, patients struggle with the medically unexplained nature of the syndrome [6,7]. In the case of many illnesses, diagnosis can serve to give meaning to an illness experience, but with fibromyalgia, initial relief is replaced with the realization that the diagnosis does not lead to increased understanding, treatment options, or respect from others [11-13].

This is where information might potentially play a role. Though patients with fibromyalgia have shown little long-term improvement [14], previous research has shown that becoming knowledgeable about one’s condition is an important factor in acceptance or coming to terms with pain [15], and pain acceptance is associated with less pain, disability, symptoms, mood disturbance, as well as better general health, functioning, and greater well-being (eg, [16,17]). Because fibromyalgia is a condition for which there are limited treatment options, self-management is increasingly being recommended [18].

Fibromyalgia patients consult many sources to try to understand their condition and possible treatments, including health care professionals, the Internet, health organizations, magazines, television, radio, support groups, and other people [19,20]. Fibromyalgia patients and other patients with chronic conditions may use online resources such as online discussion forums and blogs to exchange information, understand their illness, and offer social support [21-23]. Online participation may lead to benefits such as reduction of social isolation [22], patient empowerment [24], and improved psychological, social, and cognitive health [25].

Previous research has also reported that fibromyalgia patients’ information needs change over the course of the illness [26]. At first, individuals may be preoccupied with finding a cure. Searches for information on exercise, medications, and research increase over time. However, it is unclear what drives this evolution in information behavior, and moreover, what information behaviors may lead to successful self-management. The motivation for the current study was to provide insight concerning this gap. This paper explores three aspects of fibromyalgia patients’ illness journeys: (1) health management, (2) information seeking, and (3) information consumption and use processes.

## Methods

### Sample and Recruitment

Multiple mechanisms were used to recruit a convenience sample that self-identified as having fibromyalgia (*N*=23). A recruitment goal was established to recruit a sample that was diverse in terms of three characteristics: age (˂47 years and ≥47 years), illness duration (≤4 years and ˃4 years), and social media participation style (non-user/lurker, infrequent participator, active participator), with substantive representation in each of the subcategories per category. A lurker was defined as someone who read social media content but did not author content themselves, an infrequent participator was someone who authored social media, but infrequently, and a frequent participator was someone who authored social media content quite often. These definitions are based on those in previous studies, with modifications to account for participation on other types of social media [27,28].

The decision to focus on these dimensions was based on previous work that showed there was great variation in the age and illness duration of fibromyalgia patients and that social media participation style was significantly associated with other aspects of illness adjustment [29]. The age threshold was based on the mean age in prior studies [19,26], and the illness duration threshold was set in order to emphasize the first several years after onset.

The recruitment mechanisms included an email contact list from a previous survey [26], a university staff and student listserv, face-to-face support groups, health-related discussion forums, and Twitter (Table 1). In the case of face-to-face support groups, the leaders of support groups for fibromyalgia, chronic pain, and chronic fatigue syndrome were contacted, and permission was sought to visit the support groups to introduce the study and invite members to participate. The health-related discussion forums included websites such as Reddit, HealingWell, and ProHealth, which feature forums dedicated to fibromyalgia and other conditions that are often co-morbid with fibromyalgia, such as chronic fatigue syndrome. In each case, a description of the study and an invitation to participate was posted in relevant forums. In the case of Twitter, users who self-identified as having fibromyalgia were contacted and invited to participate.

**Table 1:** Recruitment mechanisms and participants recruited.

Recruitment mechanism	Participants, n
Participant pool from previous survey	4
Listserv^a^	7
Social networking sites	5
Face-to-face support groups	6
Twitter	1

^a^Includes those referred by someone on the listserv.

### Interviews

**Figure 1:**
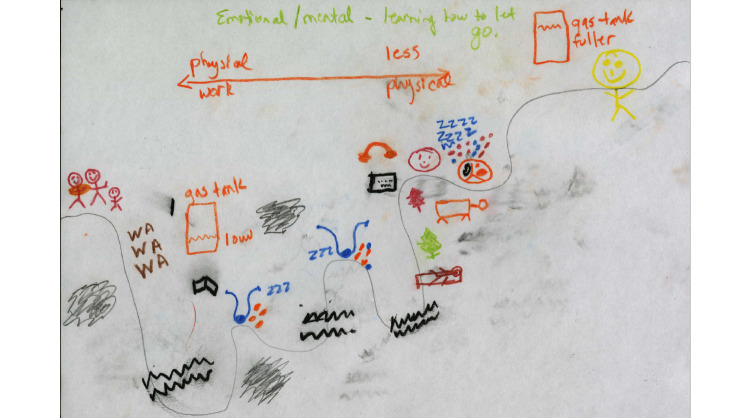
P21’s illness journey timeline.

The first interview focused on participants’ health history and information seeking and use. Participants were also asked to draw a timeline representing their illness journey (Figure 1). Timelines have been used in previous health-related research (eg, [30-32]). When the exercise was introduced, participants were asked to think about their illness journey and “to draw something that represent[ed] it.” They were told there were no rules as to what they drew and that the timeline need not be a line. The aim of the prompt was to leave the activity as open as possible, so that participants would feel free to depict the journey as they experienced it. The purpose of this activity was to help participants access their memories of their illness history.

The second interview was used to explore participants’ social media participation histories, using an interface called the Online Scrapbook that was designed for the study. This interface enabled participants to view their social media participation over time. There were multiple reasons to incorporate the interface, including reminding participants of what they had previously authored, as well as providing them a fresh look at it through an interactive visualization. As this paper focuses on thematic analysis of the interview content, the interface will not be discussed in further depth. The interview guides for the two interviews have been included in the Multimedia Appendices 1 and 2.

Participants were interviewed either once or twice, depending on the extent to which they participated in social media and their geographic proximity. If participants participated only minimally in social media or lived far away, they were usually interviewed once, and the questions from the second interview were integrated into the first interview. Three interviews were conducted via Skype or phone due to issues of geographic proximity. All other interviews were conducted in person. To ensure that participants were comfortable during the interview, the location for the interview was left up to the participant, and almost all interviews occurred either in participants’ homes or in coffee shops. Altogether, the study involved 37 interviews with 23 participants, and the mean total interview time per participant was 2 hours and 26 minutes.

### Data Analysis

The analysis method was derived from two approaches: interpretative phenomenological analysis and constructivist grounded theory. The primary aim of interpretative phenomenological analysis is to explore how participants make sense of their world and focuses on participants’ interpretations of the object or event [33,34]. Grounded theory focuses on how social and psychological processes occur in a given environment or situation [35,36]. These two foci facilitated a study that investigated lived experience as well as social interactions and context.

The interview transcripts and a purposively sampled subset of posts authored by participants who engaged in online discussion forums such as Reddit served as the basis for the analysis. Because some participants were extremely prolific in their social media content production and it was not possible to manually analyze all of the posts, it was necessary to select a subset of posts that provided a sense of the diversity of each participant’s social media production. The posts that participants authored were analyzed in the context of the threads, or dialogues, in which the posts were embedded.

The content was analyzed using Atlas.ti Version 1.0.1. In order to protect the identities of the participants, each participant was assigned an identification number. There were four pilot participants; thus, the 23 participants in the study will be referred to as P05-P27.

The analytic procedure involved initial line-by-line coding, followed by conceptualization of codes as a nested hierarchy, as is customary in grounded theory [37]. In interpretative phenomenological analysis, a similar process is performed in which the codes are thought of as belonging to themes and subthemes [33]. The themes and subthemes relating to health management and information behaviors are reported in this paper.

Exact prevalence of themes has not been reported in this study for two reasons. First, due to the sample size and recruitment strategy, the sample is not necessarily representative of fibromyalgia patients as a whole. Second, though prevalence of a theme may be an indicator of its significance, simply because a behavior occurs often does not immediately render it important [38]. However, it is understandable that a reader would like to acquire a sense of how common a behavior is from the reading, and thus, consistent conventions of “a few,” “some,” “many,” “almost all,” and “all” have been used, as in previous research [38].

The study protocol was approved by the Institutional Review Board at the University of North Carolina, Chapel Hill. All participants gave written informed consent for their data to be used in publications.

## Results

### Participants

The sample included 23 individuals who self-reported that they had fibromyalgia. The majority of the sample was white women (Table 2). The participants resided in nine different states; Washington, DC; and Australia. Because recruitment occurred using multiple mechanisms including several social media channels, the sample was naturally geographically diverse. The use of multiple recruitment methods also led to a sample that varied in terms of age (range 21-79 years), illness duration (1-58 years), and social media participation style. Though diversity was achieved in all three target categories, those with short illness durations were underrepresented.

The sample was highly educated, with the majority holding at least a 2-year or 4-year degree, and approximately half holding graduate degrees. Potential reasons for this bias were that a university listserv was used for recruitment and that those who had graduate degrees might have had a greater appreciation for the contribution of research to health care and thus volunteered for the study.

### Health Management Features of the Illness Journey

This section focuses on the health management aspects of the journey in three parts: (1) moving from onset to diagnosis, (2) acceptance, and (3) development of an acceptable level of self-management.

#### Moving From Onset to Diagnosis

Though for some participants, fibromyalgia onset coincided with an event such as a surgery or immunization, a more common pattern was experiencing symptoms for some time, before recognizing that the symptoms were not to be ignored. Most participants were diagnosed years after onset. There were various reasons. In many cases, participants did not seek medical assistance right away. P09 experienced fatigue for years, but she did not seek help because she thought she was just being lazy. It was not until she started experiencing pain that she sought the opinion of a physician. P27 had a fast-paced lifestyle full of events that she was committed to, so she ignored her symptoms until “[her] body was forced to stop.” In retrospect, she said:

Thinking back on how long I’ve been feeling a little bit tired or feeling a little bit achy and thinking of all the ways that I made excuses for that, I realized that probably my symptoms have been going on a lot longer than I thought…But I just said, well this is what it’s like to be an activist…you’re just always tired because you’re always doing stuff for the movement or for the community, so just push through it.

P27

There were other reasons why diagnosis took a long time. Physicians tended to diagnose the condition after excluding other possibilities, so many participants experienced a period of uncertainty in which they saw multiple health care practitioners and underwent many lab tests before being diagnosed. P05 remarked somewhat facetiously, “It was really crazy…a lot of ER visits, um, got to know doctors very well—all kinds of specialists…and I sit there, and I was like, I should have been a doctor. Because at this point, I have done almost every test you can think of.”

Participants’ responses to diagnosis varied, but many did not want to be diagnosed with fibromyalgia due to their impressions of the condition. P15 said of the moment when her physician made her preliminary diagnosis:

I remember thinking, “No!” That’s one of the things I never wanted to have, because…it’s like, Chronic Fatigue Syndrome or back problems…you can’t really see it, and nobody believes it’s real. And it’s one of those things that makes people out all the time from work, and…people think you’re faking it…I thought, “Oh God! Of all things!”

P15

A few participants mentioned that their mother or other relative had fibromyalgia or chronic pain, and the possibility of a genetic basis to fibromyalgia has been hypothesized in previous literature [39]. P20 remarked that she had not really believed her mother: “I thought that something had just happened to my mom, that she was making it up. Because this was in the ‘70s, when she was diagnosed with fibromyalgia. And I’m like, what kind of made up stuff is that?” So when P20 was diagnosed, she thought: “Please don’t give me that. Anything but that.”

Being diagnosed with fibromyalgia could be bittersweet. At 24, P11 thought that she might have had fibromyalgia and went through the next several years having lab tests and seeing different doctors. In a Reddit post, she wrote of her diagnosis at 30: “For me, it was relief, to finally have an answer (an answer I thought was RIGHT, as I’d thought it was fibro for a while but had never brought it up) but also a bit sad as I’m stuck with this for the rest of my life!” P24 also went through a period of frustration at the lack of answers, and she said that when she and her physician finally found a treatment regimen that worked, “that visit with her was kind of bittersweet because it was like, great, something’s working, I have an answer—but it’s fibromyalgia, and I’m going to have this for the rest of my life.”

The path to diagnosis was often long and stressful. Nevertheless, being diagnosed was important because the diagnosis enabled participants to move forward in terms of figuring out how to manage their condition.

#### Acceptance

After participants were diagnosed, it often took time for them to move towards acceptance, which consisted of two parts: acceptance of the diagnosis and acceptance of the illness. In terms of the diagnosis, participants seemed to come to accept it because their symptoms matched clinical descriptions of fibromyalgia. P10 initially did not believe that she had it, but “as time progressed, and I had other symptoms, like migraines…that went along with fibromyalgia, as I read more about fibromyalgia, I kind of accepted that I had fibromyalgia.”

The second part involved an acceptance of the illness as being there to stay. In the beginning, some participants felt that the illness was temporary, and they were looking for a way to “fix it,” for the “magic pill” [P05] or the “magic bullet” [P17]. P15 described her experience:

2013 was going to be my year of…health…it was going to be the year that I got myself back. Yeah right…at a certain point, I adjusted to a year of concentrating on wellness, instead of just, “Oh, I’m going to be…cured.” Because I kind of felt like, “I’m going to cure myself!”…I think it’s kind of like a pipe dream that some people like me will cling to and…I need to accept…I think you have to grieve like you have to grieve any other loss or death…you have to go, “Okay that’s the old me,” and “this is the new me.”

P15

#### Self-Management

Achieving an acceptable level of self-management was often multidimensional, including both symptom and emotion management, and was predicated upon acceptance:

For once in a very long time, I felt like I could handle the fibromyalgia...I was starting to realize: ok, it’s a part of my life, and I started noticing some of the triggers a little bit better, also the best way to manage some of the symptoms, and also not being so mad at myself or my body.

P05

The concept of self-management does not mean the elimination of symptoms, but rather, reaching a point where patients believe that they are able to manage their symptoms or that the extent to which they experience symptoms is “acceptable.” For many, this may mean “wellness.” Patients may still experience “flares,” but to a lesser extent. The level of functioning for each individual might differ significantly, but there is a pragmatism to it in the sense of finding solutions that fit people’s lives: “…basically what I have learned is that you just manage your life” [P21].

All participants made changes to their lifestyles. Many made dietary changes to avoid trigger foods; others had strategies such as having nuts on hand to avoid hypoglycemic episodes. Many found that exercise was helpful, particularly yoga. Participants also used alternative therapies such as massage therapy, acupuncture, meditation, and hypnotherapy. Participants reported that meditation and hypnotherapy were effective for both pain management and emotion regulation.

P21’s timeline aptly illustrates the main health management features (Figure 1). She started out her journey with a gas tank that was always low, meaning that she was constantly fatigued and struggling with different health issues. Along the way, she saw multiple doctors (represented by the stethoscopes). Towards the end of her journey, she encountered and tried multiple alternative modalities (represented by the trees). At the end, she developed an effective management strategy and was able to consistently maintain a fuller gas tank.

### Information Seeking

In terms of information seeking, the predominant pattern was a move from active information seeking to monitoring information sources with intermittent focused searching.

**Table 2:** Participant characteristics.

Characteristic	Category	n	%
**Age**			
	21-30	4	17.4
	31-40	4	17.4
	41-50	2	8.7
	51-60	7	30.4
	61-70	5	21.7
	71-80	1	4.3
**Gender**			
	Female	22	95.7
	Male	1	4.3
**Race/Ethnicity**			
	White	20	87.0
	Black	2	8.7
	Asian	1	4.3
**Education**			
	Some college	2	8.7
	2-year or 4-year college degree in progress	1	4.3
	2-year or 4-year college degree	8	34.8
	Graduate degree	11	47.8
	Graduate degree in progress	1	4.3
**Employment status^a^**			
	Student	3	13.0
	Employed full-time	11	47.8
	Not employed	1	4.3
	Retired	9	39.1
**Received disability**			
	Yes	8	34.8
	No	15	65.2
**Illness duration**			
	≤4 years	3	13.0
	˃4 years	20	87.0
**Social media participation**			
	Non-user/lurker	9	39.1
	Infrequent participator	5	21.7
	Frequent participator	9	39.1

^a^Participants may belong to more than one category.

#### Active Information Seeking

In general, participants engaged in active information seeking toward the beginnings of their illness journeys. Prior to knowing what they had, participants looked for conditions that had similar symptoms; others used symptom checkers. Some participants suspected that they had fibromyalgia and discussed it with their doctors. Participants employed a diverse array of information sources including print (newspapers, magazines, books) and digital media (Twitter, informational websites, discussion forums, blogs, webinars, e-newsletters, and e-books), people (health care practitioners, authors, family, and friends), informational and emotional support venues, patient education courses, and others (commercials, radio, and television).

**Figure 2:**
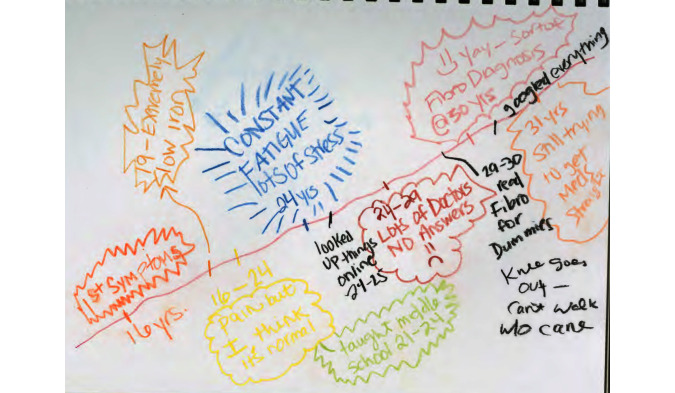
P11’s illness journey timeline.

Once participants had an idea that fibromyalgia was what they had, they would engage in more extensive information seeking. When P17 was first diagnosed, she read “everything [she] could get her hands on.” Prior to her diagnosis, P11 sought answers both online and through her doctors, but after she was diagnosed, she “googled everything on creation” (Figure 2).

A period of confusion often followed the diagnosis as participants searched for information and found no clear answers. Many participants said that they saw multiple doctors and had numerous lab tests. P19 had “this battery of tests for, you know, we call it the symphony of catastrophic diseases? Lupus, MS…” P05 said that one doctor thought it was fibromyalgia; another said arthritis, and “they’re like, we’re not exactly sure. So it was a very uncertain time in terms of figuring it out because it wasn’t getting any better” [P05]. P06’s encounters with health care practitioners did not appear to be leading towards a resolution. Thus, she ended up trying to figure things out herself: “I was doing research on my own and realizing that there was no medical consensus about what fibromyalgia was or how to treat it, so I really ended up doing a lot of research on my own” [P06].

Some participants found the diagnosis empowering in that it enabled them to do something to help themselves. In the case of P09, it helped in her search for information: “Once I had a name, I searched for fibro communities. Before that, I kind of trawled the Internet looking for places where other people talked about it…I searched for the symptoms, ignored what the forum was, and just sort of talked in various forums.” Participants found it frustrating to not know what was happening with them, and active information seeking often continued through the point of diagnosis, until they developed an effective management approach.

#### Transition to Monitoring

Eventually, study participants came to accept their illness and learned to manage it. Along with these changes, participants also engaged in less information seeking. There were various reasons. First, because information seeking was often symptom-driven, as participants were able to get their symptoms under control and/or learned to manage their condition better, they felt less of a need for information. When asked if she currently searched for information, P09 responded: “The symptoms fit the diagnosis, and while it’s frustrating to have that diagnosis and I don’t like it, and I wish it were something that were curable, I’ve mostly stopped, um, because the current treatment regimen is helping.” Many participants reached a point where they just wanted to move on:

For me, you just kind of reach a point where it’s like (sigh), “It is what it is. I’m going to continue to eat as healthy as I can. I’m going to continue to walk every day. I’m going to continue to lead as healthy a life as possible.” But I really am kind of done trying to figure it all out. I just want to get on with my life…When I have a bad day, “Oh well,” I don’t really question it anymore.

P14

Participants also stopped seeking information because they felt that they knew what was out there, and there was no new information to be found. P06 said, “I occasionally get back online and kind of look up and see where the research is at. Every now and then I’ll see a research study highlighted about fibromyalgia and I’ll read it just to see if there’s any major breakthroughs [chuckle], but there never are.” Most participants tried to maintain some level of awareness of fibromyalgia research through “monitoring,” that is, subscribing to e-newsletters from services such as Medscape. There is a lingering sentiment of wanting to know: “I’m still signed up for a couple of newsletters, but I don’t look at them on a regular basis. They flood…they come to my inbox and I don’t want to…unsubscribe…there are moments…I just want to see latest conversations, and latest research...So I keep them…they all go into a folder” [P05].

Searching for information on an as-needed basis also served to reduce information overload: “[I] started looking for specific things for specific problems…I wanted to piece [together] what would cause different areas of the symptoms, instead of looking at it as a whole, ‘cause then it was just overwhelming” [P05]. Thus, on the whole, participants eventually settled into a pattern of monitoring. But once in a while, the appearance of new symptoms, serendipitous encounters with new information, and other events might trigger some focused searching.

If a patient develops a new condition, they may cease monitoring and cycle back to active information seeking. For example, after P17 developed fibromyalgia, she engaged in active information seeking and participated in online discussions, but eventually her participation waned. She started seeking information and participating again, after being diagnosed with alopecia.

### Information Consumption and Use Processes

Participants engaged in information consumption and use processes throughout the course of their journey. Several key processes emerged: forming a coherent representation of the conceptual space, evaluation and synthesis of information, taking charge of one’s health care, and iterative problem solving.

#### Forming a Coherent Representation of the Concept Space

Over time, participants came to understand the concept space in different ways. P05 became acquainted with the scientific explanation: “I know the biology and the science behind fibromyalgia, what they say about it, the causes they don’t really know about it…” Many came to know fibromyalgia in terms of the symptoms: “Mostly when I was still learning what fibromyalgia is, I was looking at symptoms and stuff like that… at all these different websites explaining what is going on, and what is its effect, and how people with fibromyalgia are going to feel” [P13]. P06 surveyed the online space and selected treatments based on her own comparison of patient reports: “I felt like I was doing all the major recommendations…massage, getting a lot of rest, the one particular drug that a lot of people have had success with, the guaifenesin.” There are differences in the ways that participants represented this space, but each formed an understanding of the space that they could accept, in other words, that was coherent to them.

At some point in their illness journeys, many participants came to a point of saturation, where they felt as if there was “nothing new” [P20] and that they knew “all there was to know” [P25]. This coincided with the transition to monitoring described earlier.

#### Evaluation and Synthesis of Information

As they were coming to understand the concept space, participants continually encountered and evaluated information. Many participants read extensively about fibromyalgia and synthesized across sources. They developed their own heuristics for evaluating the quality of information. One common rule was that they dismissed information that “promised a cure” [P09]. P22 looked “to see if they’re accurate about the basic mechanics of how it [fibromyalgia] works.” Others looked for consensus across multiple sites.

P13 and P27 engaged in another type of synthesis, involving comparison of explanatory perspectives on fibromyalgia. P13 quickly realized that allopathic medicine’s explanation of fibromyalgia did not satisfy him and moved on to study Chinese medicine. P27 said, “As soon as the doctors are thinking, okay, this might be fibromyalgia, I started doing research on the Internet, but then also checked out at least ten fibromyalgia books from the library, just to read different perspectives on what fibromyalgia is and, like, differing ways that you can treat it.”

#### Taking Charge of One’s Own Health Care

Over time, many participants realized that they needed to take charge of their own health care. They showed this initiative in various ways. P15, like many others, went through a prolonged period of lab tests and consultations prior to being diagnosed. During that time, she realized that even if she were diagnosed, she would refuse medication, so she needed to take matters into her own hands: “I was not getting what I wanted to get with the doctors and all that, so I thought, ‘Well, what would you do differently if you had the diagnosis?…Whatever that is, you need to start doing it now’” [P15]. Then she began an elimination diet, which involved progressively removing items from her diet until she figured out what she was sensitive to.

Though health care practitioners may be experts in their respective areas, patients are likely to have a more intimate understanding of their own body. P12 explains the rationale for taking charge: “Doctors know some things, and you know some things. And you’ve got to have somebody who lets you put that together, ‘cause you’re the expert on your body, and they might be the expert on some treatments, but then you’re the one that has to sort of be your own case coordinator, and monitor your body.”

**Figure 3:**
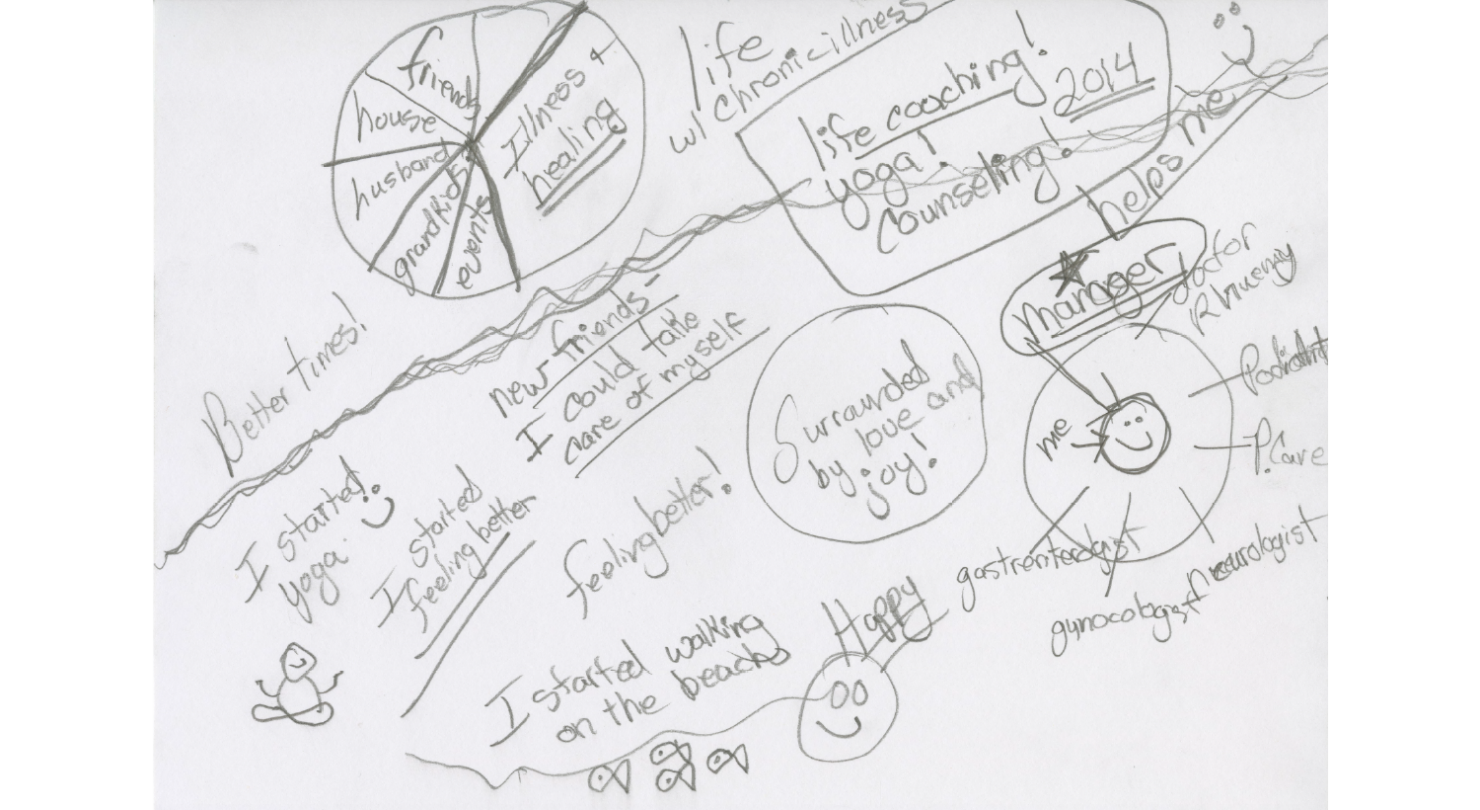
P14’s illness journey timeline.

This was not a role that participants naturally took on: “It took me a long time to be the manager of my own health system. I expected doctors to kind of manage my life for me. It took me a long time to realize that, no, I’m in charge of this. The doctors that work for me are a team, and I manage that team” [P14]. P14 depicts herself as a manager of her multidimensional care team (Figure 3, right).

#### Iterative Problem Solving

Over the course of their illness, participants also engaged in episodes of problem solving to address the physical problems that they experienced.

Figuring out a management strategy was often an iterative process involving trial and error. For example, in the case of exercise, participants often needed to experiment to find the right type of exercise and amount that they could handle. P17’s story is a case in point: “I got myself an exercise bicycle…I worked up to ten minutes a day…but then the other 24…23 hours a day I was in the bed. So I figured that’s not going to work.” She said that eventually, “I learned to evaluate myself, how tired I was getting, and how I was feeling, so I could better pace myself and rest when I needed to, and that made a huge difference...And yoga helped a lot in helping me become self-aware” [P17].

Another common issue was identifying food sensitivities and allergies. Some participants, such as P15, researched how to do this on their own. P24 used a mobile app called Pain Coach to track her food intake. This enabled her to figure out that gluten was causing her a problem, and then she eliminated it from her diet. P26 underwent lab tests and a rotation diet to figure out the foods to which she was sensitive.

Participants progressively made adjustments and/or engaged in additional information seeking based on evolutions in their thoughts and often, in their symptoms. P15 described this as “peeling back layers”:

Like with the nutrition, say I have an issue with certain types of foods…that’s one thing, and then I am getting better nutrition, not the processed things…more organic stuff, and that’s peeling off another layer…and getting better exercise and more movement, of the proper kinds of movement that don’t cause me to have pain. That peels off another layer. And that exposes something else.

P15

Not all of participants’ energies were engaged in problem solving via information seeking; there was also internal sense-making. Participants found it frustrating that there were so many unexplained symptoms, and they were constantly trying to figure out the root cause of their problems: “If you have any intelligence at all and you want to get better, you want to try to figure out why you woke up feeling so bad. So I would go back and go through all the foods that I ate and go through everything. Did I go through these stresses?” [P14].

## Discussion

### Principal Findings

This paper reported the findings of a qualitative study of fibromyalgia patients’ illness journeys. At the outset, there were health-related features: moving from onset to diagnosis, acceptance, and development of an acceptable level of health management. Information seeking changed over time, particularly in terms of a transition from active information seeking to monitoring. Last, patients engaged in information consumption and use processes: forming a coherent representation of the conceptual space, evaluation and synthesis of information, taking charge of one’s health, and iterative problem solving.

**Figure 4:**
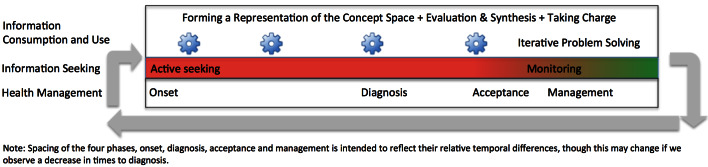
The relationship of information behavior and health management over time.

Aligning the themes from the interview content temporally affords an integrated conceptualization of how health management and information behaviors are related over time (Figure 4). Patients engage in active information seeking at the beginning of their journeys, beginning with cognizance of their condition and extending through diagnosis and acceptance. As they begin to develop an acceptable level of management, their information seeking tapers to a pattern of monitoring with intermittent focused searching. The spacing of the four phases (ie, onset, diagnosis, acceptance, and management) is intended to reflect their relative temporal differences, though this may change if we observe a decrease in times to diagnosis.

Alongside these developments, participants continually engage in information consumption and use. They form and refine their interpretations of the concept space. They also engage in information evaluation and synthesis activities, which become more sophisticated over time but decrease due to lessened need. They learn to take charge of their health care. Periods of iterative problem solving to address issues such as irritable bowel, fatigue, and sleep problems also occur. The information consumption and use processes might also be thought of as activities that individuals become increasingly skilled at over time. A set of arrows from monitoring back to active information seeking indicates that patients may traverse the process again for a new condition.

The journey described in this paper reflects the predominant patterns among study participants. Though most participants engaged in extensive information seeking, a few participants did not. P18 said, “I didn’t really look into it [fibromyalgia]…I just kind of accepted it for what it was and didn’t worry about it.” P07 said that she was never really a very curious person. Both participants ended up learning more about fibromyalgia serendipitously, decades after they were diagnosed, when they engaged in exercise that inadvertently led to health information encounters. For P10 and P25, who had conditions that pre-dated fibromyalgia, there also seemed to be less of an impact.

### Comparison With Prior Research

Though there has been considerable work investigating the lived experiences of fibromyalgia patients (eg [5,40-42]), at least to this author’s knowledge, there is no existing work that has taken a qualitative approach to investigate the relationship between health management and information behavior in fibromyalgia patients over time. The results of this study illustrated that, over time, participants became clearer and more accepting of their condition. These developments were accompanied by an increased awareness of and ability to use information sources to improve their health management, as well as improved communication with physicians and other health care providers.

The study findings share similarities with existing research in health literacy and self-management in chronic illness. In this study, participants developed skills that have also been identified in a meta-synthesis of self-management in chronic illness (eg, taking ownership of health needs, adjusting to illness, and activating resources) [43]. Additionally, the study findings showed that participants developed skills that have been associated with increased health literacy, such as becoming more active in clinical consultations, and greater autonomy and empowerment in decision making [44-46]. Previous research testing an Internet-based self-management intervention for fibromyalgia has also demonstrated that increased knowledge can lead to better health outcomes [47]. This convergence in the study findings and the self-management literature suggests that some of the lessons learned from this study might be incorporated in the design and delivery of self-management education.

### Implications for Information System Design

There are various ways that information technology could support fibromyalgia patients’ information needs. First, to make sense of their condition, participants attempted to synthesize information across diverse source types and from multiple explanatory perspectives. Because they are not focusing on a single information source, tools that help patients make sense of and compare information sources could be particularly helpful. Building interfaces that enable patients to discern and make sense of explanatory perspectives has also been suggested in a previous study concerning information about Lyme disease [48]. Systems that help users understand and evaluate health information from different perspectives could be invaluable for conditions in which there are many treatment options, multiple alternative perspectives, and unclear treatment protocols.

Participants also expressed frustration because there were so many factors that could be influencing their health, and it was difficult to disentangle them (eg, P14). Thus, the development of tools to analyze different types of personal health data and the integration of knowledge bases to provide additional information are important directions for future development. Fibromyalgia patients are also more likely to have comorbidities [49], which can make it seem like work to track data [50].

Last, once patients start developing a picture of the concept space, they experience less of a need to seek information but are still interested in keeping up with the research. Patients who engage in monitoring could benefit from tools that automatically process newsfeeds and flag articles for perusal.

### Limitations and Future Directions

Though this study provided valuable knowledge about how people use information in the context of chronic illness, there are limitations. The participants in this study had all reached acceptance, most had achieved a stable level of health management, and almost all had stopped actively seeking information. As such, the study afforded a glimpse of participants’ journeys through their eyes, at a particular point in their journeys. There are potential problems with retrospective recall, including errors in memory, lack of clarity about past events, and differences in interpretation of one’s history over time. Some persons may never reach acceptance or develop effective management strategies; their perspectives are not reflected in this sample.

Thus, an important priority for future research is to work with patients earlier in their illness journeys. Though participants in this study reported developing a more coherent representation of the concept space, more familiarity with and ability to evaluate information sources, and increased ability to take charge of their health care over time, there is still much that we do not know about how these skills evolve. Additional research focusing on critical time periods such as postdiagnosis, and important activities, such as problem solving and sense-making about symptoms, could inform the design of patient education programs.

## Appendix

Multimedia Appendix 1 Interview 1 Guide.

Multimedia Appendix 2 Interview 2 Guide.
