# Correction: The Relationship Between Health Management and Information Behavior Over Time: A Study of the Illness Journeys of People Living With Fibromyalgia

**DOI:** 10.2196/23597

**Published:** 2020-08-20

**Authors:** Annie T Chen

**Affiliations:** 1 University of Washington School of Medicine Department of Biomedical Informatics and Medical Education Seattle, WA United States

In “The Relationship Between Health Management and Information Behavior Over Time: A Study of the Illness Journeys of People Living With Fibromyalgia” (JMIR 2016;18(10):e269) the author noted an error in Table 2. Some values under the section “Employment status” were listed incorrectly.

Values for “Student” were originally listed as:

n=11, 47.8%

The correct values for “Student” are:

n=3, 13.0%

Values for “Not employed” were originally listed as:

n=3, 13.0%

The correct values for “Not employed” are:

n=1, 4.3%

The correction will appear in the online version of the paper on the JMIR Publications website on August 20, 2020, together with the publication of this correction notice. Because this was made after submission to PubMed, PubMed Central, and other full-text repositories, the corrected article has also been resubmitted to those repositories.

