# Supplementary material for: The Relationship Between Health Management and Information Behavior Over Time: A Study of the Illness Journeys of People Living With Fibromyalgia
Source: J Med Internet Res. 2016 Oct 25;18(10):e269. doi: 10.2196/jmir.5309 (PMC5101414; doi:10.2196/jmir.5309)
Supplement: Multimedia Appendix 1 [file jmir_v18i10e269_app1.pdf]

Appendix 1 Interview 1 Guide

<u>Introduction (20 min.):</u>
The researcher performs a self-introduction, goes over the consent information with the participant, and asks the participant to fill out background questionnaires.

<u>Opening questions (10 min.):</u>
Now let's proceed with some questions about your health history.

1. Can you tell me when your health issues first began?
2. (probe further) Can you tell me more about that?
3. How did you feel at the time?
4. What sources did you go to for help? (This can refer to medical help, social support, or information.)
5. How were those initial experiences?

<u>Timeline Activity</u>

At the start of the activity, the researcher asks:

I'd like to ask you to think back to when you started experiencing health issues, or further, if you think it's relevant. Think about the path, the journey you took to get to where you are today, and draw something that represents it.

There are no specific rules about how your drawing should look; you can be free with the form it takes. It can have a line, or not have a line.

You can use whichever colors you like, or just one color, if you want. You can use different colors to represent different aspects of your experience.

If you need more paper, we can attach an extra sheet.

Please talk aloud as you are drawing the timelines. You can just talk to me about what you are drawing, why you are drawing it, anything that occurs to you. The purpose of me asking you to talk aloud is to help me understand what matters to you, and also to understand your memories as you do. (Prompt the participant if he or she falls silent.)

Some possible probes include:
Could you tell me about what happened then?
Could you tell me about what you're drawing now?
Could you tell me about that change?

Suggest that the participant add the following to the timeline:
Emotions
Turning points
Gradual changes (e.g., periods of frustration, periods when you felt that things were going well) Important events ("What would you say is an important event?")

Ask the participant:

1. Thinking back to your illness journey, at what points do you think information came into play? Can you note these points on the timeline?

2. (probe) In what ways did information come into play?

3. Throughout your illness journey, what information was most important to you?

4. Did you look for information? (If yes, why?)

5. Did you receive information from practitioners or people around you?

6. How did you feel at these times? (Frustrated? Hopeful?)

7. Do you remember anything that you learned, or any information that you received, that changed the way you viewed your illness, your health or your life?

8. Do you remember anything that you learned, or any information that you received, that changed the way you do things or manage your illness?

9. From what sources did you receive information? Have these changed throughout the time that you have had your condition(s)?

(After the participant is done, ask them some questions to try to clarify points that may be unclear, if any, on the timeline.)

Follow-up questions (30 min.):
Information Behavior
1. Do you feel that you have generally received the information that you needed to know how to deal with your condition?
2. Today, do you feel that you still need information about your condition, and if you do, how do you go about finding what you need? (Make sure that they touch upon the sources that they use, how they conceptualize the search, and their search strategy.)
    a. What sources did you use?
    b. How do you go about looking for information?

Health Management
3. Now can you tell me about how you manage your condition today?
4. What do you see as your priorities?
5. Has your management strategy changed over time?
6. Do you see this changing in the future, and if so, how?
7. How do you feel about your health now?
8. What do you think that health and wellness mean?
9. Has this changed for you over time?

10. Do you feel that those around you are supportive of you?
11. Where do you personally go for support?
12. Is there anything else you want to tell me about your illness journey?

The researcher thanks the participant and concludes the interview.
