# Supplementary material for: The Relationship Between Health Management and Information Behavior Over Time: A Study of the Illness Journeys of People Living With Fibromyalgia
Source: J Med Internet Res. 2016 Oct 25;18(10):e269. doi: 10.2196/jmir.5309 (PMC5101414; doi:10.2196/jmir.5309)
Supplement: Multimedia Appendix 2 [file jmir_v18i10e269_app2.pdf]

Appendix 2 Interview 2 Guide

Thank you again for your time. I'm looking forward to our time together here today. We will begin by talking about your experiences participating in online spaces.

1. Can you start by briefly talking about the online spaces that you participate in? (Might want to probe with: "For example, do you use Twitter, Facebook, etc.?" It would be helpful to have the information from the pre-screening questionnaire on hand.) Why do you participate in online spaces?
2. What does "online community" mean to you? Has this meaning changed over time?
3. Do you obtain information from the online community you participate in? Can you give some examples?
4. Do you give information to the online community you participate in? Can you give some examples?
5. Do you feel that the members of the online community support each other? Can you give some examples?
6. What do the members of the online community mean to you? When and how do you interact with them?
7. How has your participation in online support groups changed over time? Can you give some examples?

(If, in answering the questions above, the participant has not touched upon multiple communities/platforms, but their pre-screening questionnaire indicates that they do participate in multiple media, it would be good to ask them about that.)

Online Scrapbook Viewing Activity

In the last session, you agreed to let me collect some of the content that you've authored online. Now I'd like to show you some of this content, and for us to explore it together. Okay? (Wait for the participant to agree.)

(Access the *Online Scrapbook* application.) This is an application that I've developed for this study. It's called the Online Scrapbook. Here's how it works:

At the top, you'll see there is a timeline. There is one row for each social media type that you told me about in your pre-screening questionnaire. There is a circle for each month that you participated; the bigger the circle, there more you participated during that time. If you click on the circle, you can see all of the content you authored during that month.

You can also search for your posts that contain a certain keyword. So if you think of something that you might have written about, that you want to tell me about, you can type the keyword in the box and hit the "Go" button.
Would you like to explore? (Hand the computer over to them and see how they interact with it.)

Here, it would be useful to see what they might naturally do with the interface before trying the probes below:

1. Is there anything that stands out to you about this timeline? (Ask them to elaborate on the reasons why the artifacts stand out to them.)
2. Are there any pieces that were particularly meaningful for you when you wrote them? Why?
3. Are there any pieces that are particularly important to you now? Why?
4. Are there any topics that you remember writing a lot about in the past? Why?
5. In the past, when you have written of your own experiences, have you had any responses back that were particularly meaningful to you?
6. Depending on the situation, did you tend to use different types of social media to communicate with others?
